# Suprarenal Masses in Very Young Infants: Is It Safe to Watch and Wait? Report of a SIOPEN Observational Study Results

**DOI:** 10.3390/cancers14164007

**Published:** 2022-08-19

**Authors:** Vassilios Papadakis, Vanessa Segura, Massimo Conte, Dominique Plantaz, Andrea Di Cataldo, Gudrun Schleiermacher, Kate Wheeler, Jose D. Bermúdez, Shifra Ash, Bénédicte Brichard, Ruth Ladenstein, Valérie Combaret, Sabine Sarnacki, Anna Maria Fagnani, Claudio Granata, Adela Cañete

**Affiliations:** 1 Department of Pediatric Hematology-Oncology, Aghia Sophia Children’s Hospital, Levadias Street 8, 11527 Athens, Greece; 2 Instituto de Investigación Sanitaria La Fe, Fernando Abril Martorell 106, 46026 Valencia, Spain; 3 Department of Hematology-Oncology, Giannina Gaslini Children’s Hospital, Via Gerolamo Gaslini 3, 16148 Genoa, Italy; 4 Department of Pediatrics, University Hospital Centre of Grenoble, Av. des Maquis du Grésivaudan, 38700 La Tronche, France; 5 Department of Clinical and Experimental Medicine, Unit of Pediatric Hematology and Oncology, University of Catania, Piazza Università, 2, 95124 Catania, Italy; 6 Siredo Pediatric Oncology Center, and RTOP (Recherche Translationelle en Oncologie Pédiatrique) U830 Inserm, Institut Curie, 26 rue d’Ulm, 75005 Paris, France; 7 Department of Paediatric Haematology and Oncology, Oxford Children’s Hospital, Headington, Oxford OX3 9DU, UK; 8 Department of Statistics and O.R., University of Valencia Av. de Blasco Ibáñez, 13, 46010 Valencia, Spain; 9 Pediatric Hematology Oncology and Bone Marrow Transplantation Division, Ruth Rappaport Children’s Hospital, Rambam Health Care Campus, Efron St. 19-27, Haifa 3109601, Israel; 10 Department of Paediatric Haematology and Oncology, Cliniques Universitaires Saint Luc, Av. Hippocrate 10, 1200 Brussels, Belgium; 11 St. Anna Children’s Hospital, Department of Paediatrics, Medical University of Vienna and Children’s Cancer Research Institute, Department for Studies and Statistics and Integrated Research, Zimmermannplatz 10, 1090 Vienna, Austria; 12 Laboratoire de Recherche Translationnelle, Centre Léon Bérard, Léa et Napoléon Bullukian, 69008 Lyon, France; 13 Department of Pediatric Surgery, Necker Enfants-Malades Hospital, AP-HP, Université de Paris Cité, 149 rue de Sèvres, 75015 Paris, France; 14 Pediatric Surgery Unit, Department Woman-Child-Newborn, Fondazione IRCCS Ca’ Granda Ospedale Maggiore Policlinico, Via Commenda, 10, 20122 Milano, Italy; 15 Department of Paediatric Radiology, IRCCS Giannina Gaslini Children’s Hospital, Via Gerolamo Gaslini, 3, 16147 Genoa, Italy; 16 Pediatric Oncohematology Unit, University and Polytechnic la Fe Hospital, Department of Pediatrics, Fernando Abril Martorell 106, 46200 Valencia, Spain; 17 Facultad de Medicina, Universidad de Valencia, Av. de Blasco Ibáñez, 15, 46010 Valencia, Spain

**Keywords:** suprarenal masses, neuroblastoma, observation, surgery, infant, neonate

## Abstract

**Simple Summary:**

Optimal management of small suprarenal masses (sSRMs) is not clearly defined in the literature. Among the differential diagnosis of these sSRMs without a clearly defined clinical management, neuroblastoma is the malignant neuroblastic tumor, with very good prognosis in most cases at this age and a very intriguing biology. The concept of the sSRM study is to attempt to safely minimize invasive procedures (including surgery) without jeopardizing the final outcome. We report the first International Society of Paediatric Oncology European Neuroblastoma (SIOPEN) cooperative prospective study of expectant observation as primary approach for neonates and infants less than or equal to 90 days of age with small localized suprarenal masses. In most cases, patients avoided surgery and, consequently, morbidity and mortality related to surgery. The study contributes to improving knowledge about the natural history and biology of neuroblastoma during early infancy.

**Abstract:**

**Background:** To assess whether expectant observation of infants ≤ 90 days old with small suprarenal masses (sSRMs) could avoid unnecessary surgery without impacting outcome. **Methods:** Infants ≤ 90 days with a ≤ 5 cm mass, without midline extension or lymph node or distant spread were registered (ClinicalTrials.org:NCT01728155). Once staging was completed, they were followed with ultrasound, MRI and urinary catecholamines. Surgical resection was only planned if there was a ≥40% mass volume increase or for a mass persisting after 48 weeks of the planned observation. **Results:** Over a 5-year period, 128 infants were registered. No infant had detectable MYCN amplification in the peripheral blood. Surgery was performed in 39 (30.5%) patients, in 18 during and in 21 after the planned 48-week observation, and 74% were confirmed to be neuroblastomas. Non-life-threatening surgical complications occurred in two cases. The 3-year overall survival and event-free survival were 100% and 87.1%, respectively. The 16 events observed were volume increase (N = 11) and progression to neuroblastoma stage MS (N = 5). Patients with solid masses or MIBG-positive masses had lower EFS. **Conclusions**: Expectant observation for infants with sSRMs with clinical follow-up and timely imaging (including MRI scan) is safe and effective, allowing surgery to be avoided in the majority of them.

## 1. Introduction

The incidence of small suprarenal masses (sSRMs) has increased, possibly due to the increased use of routine prenatal ultrasonography (US). According to the massive screening studies reported in the literature, it is known that these masses are usually discovered during a routine obstetric ultrasound examination or during one in the first 3 months of age, usually performed for other pediatric diagnosis or incidental finding (i.e., hydronephrosis, palpable abdominal mass). Clinical management remains controversial with neuroblastoma being the most frequent diagnosis [1,2,3,4,5]. The prognosis is usually very good, based on the predominantly favorable biological profile and the capacity for spontaneous regression [6].

Historically, the standard approach has been immediate surgical resection. In 2011, the International Society of Pediatric Oncology European Neuroblastoma Study Group (SIOPEN) launched the “Low and Intermediate Risk Neuroblastoma” study (LINES:ClinicalTrials.org:NCT01728155) including management of sSRMs, suggesting that immediate surgery could be eliminated safely, without jeopardizing patient outcome. sSRMs can be neuroblastic tumors or adrenal hemorrhage, enteric duplication cysts, subdiaphragmatic extralobar pulmonary sequestration, adrenal cytomegaly and adrenocortical tumors. In young infants, neuroblastic tumors (neuroblastomas or ganglioneuroblastomas) have favorable biological characteristics and rarely MYCN amplification (an unfavorable feature) [7]. Careful systematic follow-up can facilitate detection of disease regression or progression (local or metastatic, including to neuroblastoma stage MS) [8]. Although the feasibility and safety of the observational approach has been studied in other cooperative groups, the current study aimed to confirm and expand the results of those preliminary studies [9,10,11,12,13,14,15,16,17,18]. In an effort to be conservative and safe in the proposed non-surgical approach to sSRMs, we opted to include in this protocol younger infants and smaller masses with benign anatomical characteristics.

## 2. Materials and Methods

### 2.1. Patients

Eligibility criteria included age ≤ 90 days, with an sSRM ≤ 5 cm in maximum diameter, not crossing the midline and without regional extension, detected by US (antenatally or postnatally), and magnetic resonance imaging (MRI) within the first 9 weeks, and no evidence of metastatic involvement. For concurrent discordant measurements (US > 5, MRI < 5 cm) the MRI value defined patient eligibility.

The protocol-mandated diagnostic procedures were: complete blood count, biochemical profile (including lactate dehydrogenase, LDH), urine catecholamines and free cortisol and plasma stored for subsequent MYCN status evaluation [19,20,21].

^123^I-Metaiodobenzylguanidine (MIBG) scan was mandatory within the first 9 weeks of age in all infants with raised catecholamines or with a lesion > 1 cm, for neuroblastoma evaluation (including metastatic disease). Bone marrow aspirations and biopsies were not required, as they are invasive and difficult procedures in neonates.

Date of diagnosis was the date of initial sSRM detection or the date of birth in case of antenatal diagnosis. Urinary catecholamine (vanillylmandelic acid (VMA), homovanillic acid (HVA), and dopamine) levels were considered to be elevated if the values were >x2 the upper normal value (u.n.v.) and were required every 3 weeks until week 12. Informed consent was provided by parents or legal guardians. The study was approved by Ethic Committees and Competent Authorities.

### 2.2. MYCN Plasma Analysis

Peripheral blood (EDTA) was collected at diagnosis, plasma was stored at −80 °C and then shipped to the reference laboratory for analysis. After circulating free DNA (cfDNA) extraction and quantification, MYCN amplification status was determined by Real Time quantitative PCR (2012–2014) or Droplet Digital PCR (ddPCR, after 2014) as published [21]. Results were not delivered in real time to the centers.

### 2.3. Management

**Figure 1:**
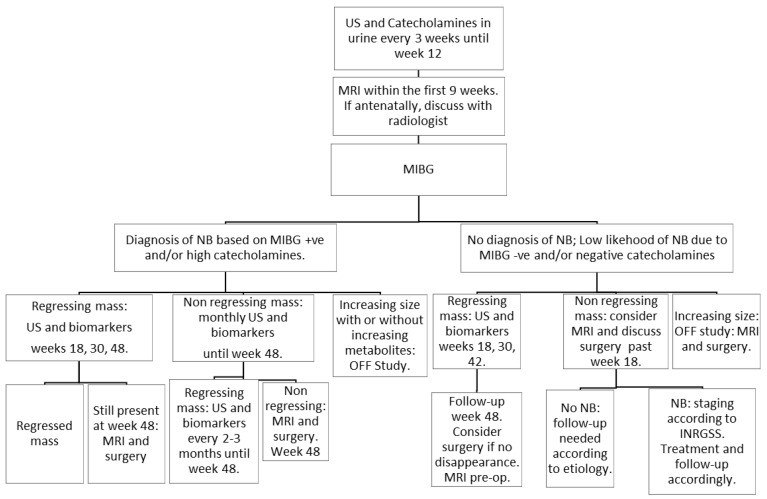
Therapeutic management of suprarenal masses with follow-up investigations at prescribed intervals during the observational course of the study.

The protocol did not indicate any pre-natal management. Following post-natal diagnosis, close monitoring was requested (Figure 1 and Table 1), without further therapeutic intervention. The follow-plan was designed to reveal early non-desirable development, indicating neuroblastoma progression. Two conditions interrupted the observation strategy and prompted surgical complete excision: (1) ≥40% increase of the original tumor volume, and (2) mass persistence following a 48-week observation period.

Isolated catecholamines increase, without increase in mass size did not prompt surgery.

Neuroblastoma restaging according to INRGSS was undertaken if: (1) there was mass regression or disappearance with persistence of elevated catecholamines, and (2) there were clinical symptoms/signs suggesting progressive disease. A 3-year annual follow-up was suggested.

### 2.4. Statistical Analyses

Due to the expected low incidence of sSRMs, a single arm design was chosen, aiming for descriptive statistics in a patient cohort recruited over five years, and estimate target accrual of 100 patients. The primary aim of the study was to maintain a 3-year surgery free survival of over 80%. Overall survival (OS), event free survival (EFS), and resection-free survival (RFS) were estimated using the Kaplan–Meier method. Pair-wise comparison was performed using log-rank tests, adjusting the *p* values by the Bonferroni method.

An event was defined as a >40% increase in mass size (volume) and/or progression to higher neuroblastoma stage (L2, M, or MS). The time to event was defined as the time from the date of diagnosis to the first occurrence of an event, or until the time of last contact if no event occurred. In RFS, the occurrence of a resection was the only event considered. Two-sided 95% confidence interval for 3-year EFS and RFS were also calculated. Cox regression assessed the combined effect of tumor nature and volume on EFS, with volume as covariate and structure of the mass (cystic, mixed or solid) as strata to ensure the proportional hazards hypothesis. A longitudinal descriptive analysis studied the mass kinetics. Time to complete regression was defined as time to complete disappearance of mass volume on imaging with normalization of catecholamines in at least two consecutive follow-up assessments.

Based on MIBG results and urinary catecholamines, the following groups were defined. Group 1 (MIBG+): Suprarenal masses with MIBG uptake; Group 2 (MIBG-): Suprarenal masses without MIBG uptake; Group 3 (MIBGND): suprarenal masses with no MIBG scan performed at diagnosis.

## 3. Results

From July 2012 to December 2015, 130 patients with detected sSRMs were enrolled in 42 SIOPEN centers in six countries. Two patients were ineligible (masses > 5 cm). Hence, 128 children (60.9% males) with median age at diagnosis of 3 days (range, 0–87) were analyzed. Patient characteristics are given in Table 2.

Fetal ultrasound (US) led to prenatal diagnosis in 54 patients (42.2%), at median gestational age 33.5 weeks (range, 18–41). The mean initial mass diameter was 2.49 ± 0.15 cm. Thirty-two (59.3%) masses were reported to be cystic, 10 (18.5%) solid and 12 (22.2%) mixed (cystic and solid components). Thirteen of 54 patients also underwent prenatal MRI.

All 128 patients had postnatal US, performed at a median age of 8.5 days (range, 0–90). The mean initial suprarenal mass diameter at the first postnatal US was 2.95 ± 0.09 cm (median value: 3.0, range, 0.8–5.9, 1 child with 5.9 cm diameter by US but 4.8 cm by MRI, was eligible). Thirty-five (27.3%) patients had cystic masses, 47 (36.7%) solid, and 46 (36%) mixed. Mass vascularization was present in 35/128 patients (27.3%), absent in 55/128 (43.0%), and not evaluable in 36/128 (28.1%). Prenatal and postnatal US studies size measurement concordance was good (Figure S1).

Eighty-two children underwent postnatal MRI, at a median age of 36 days, (range, 5–119). Median mass diameter was 2.5 cm (range: 0–5). Four masses (4.9%) detected by initial US were not evident on MRI 32 to 68 days later. Forty-six patients had no MRI evaluation before 9 weeks, despite protocol requirements, due to: 1 parental refusal, 9 physician decision (very small mass size), 3 early progressions, 11 near mass regression on US before 9-week timeline, 6 delayed to 18 week, and 16 unknown. No cases had any midline extension. By MRI 22/77 (28.6%) masses were cystic, 28/77 (36.4%) solid, and 27/77 (35.0%) mixed. There was good concordance in mass structure and size definition between US and MRI.

Patients with elevated (>x2 u.n.v.) analytical parameters values at diagnosis were: LDH (N = 91) 8.8%, VMA (N = 112) 7.1%, HVA (N = 102) 4.9%, dopamine (N = 58) 5.3%, and free cortisol (N = 31) 0.0%.

A total of 97 (75.8%) plasma samples were collected and all were negative for detection of circulating MYCN amplification. One patient with a residual mass of 1.4 cm at 48 weeks of observation underwent excision two months later and pathology confirmed an MYCN amplified neuroblastoma by FISH, with MYCN plasma being negative at diagnosis.

Seventy-five (58.6%) patients had ^123^I-MIBG scintigraphy, 54 (72%) within the protocol timeline of before 9 weeks and 21 after 9 weeks; MIBG was positive in 42 masses (56%). Five patients with increased catecholamines (>x2 u.n.v.) at diagnosis did not undergo an MIBG scan. Thyroid protection was documented in 63/75 infants, 57 (90.5%) with potassium iodide, 4 (6.3%) with iodide perchlorate (Irenat©) and in 2 cases (3.2%) it was undetermined.

Patients were divided into 3 groups according to the MIBG results: Group 1 (MIBG+) (N = 42): MIBG-positive with normal catecholamines (N = 32), elevated (N = 8) or not done (N = 2); Group 2 (MIBG- (N = 33): MIBG negative with normal catecholamines (N = 29) or not done (N = 4); Group 3 (MIBG-ND) (N = 53): MIBG not done and either normal catecholamines (N = 45), or elevated (N = 5) or not done (N = 3).

All 128 patients were followed for a median time of 38.7 months (range, 0.7–71.0) and 120 (93.7%) completed the planned observation period (48 weeks). Eighty percent (80%) of the patients completed the 3 year-follow up.

### 3.1. Events

There were 16 events during the initial observation: 11 children with tumor volume increase over 40% and 5 progressions to MS (Table 3). Three events were unusual: one mass disappeared on US at 3 months (no MRI) and at 2 years it was detected again and surgically resected (diagnosis of neuroblastoma); the second case showed a tumor increase at 26 days, which was excised (diagnosis: L1 neuroblastoma), then the patient developed bone metastases as a second event at 11 months of age and the patient is alive following 3 years of follow-up; the third case had an increase in tumor size at 43 days, this mass was completely excised (diagnosis L1 neuroblastoma) but the patient developed liver metastases 27 days later (neuroblastoma stage MS at 70 days of life).

**Figure 2:**
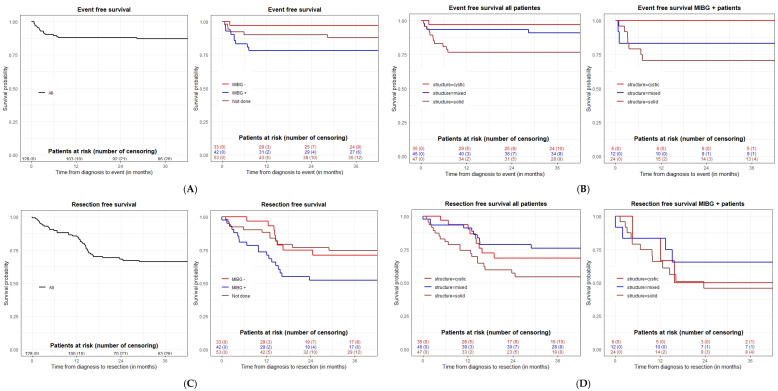
(**A**) **Left:** Event-free survival for the whole cohort (*n* = 128) patients enrolled in the study. **Right:** EFS for three cohorts of patients based on MIBG procedure: positive (Group 1, n= 42), negative (Group 2, *n* = 33) and patients with MIBG not done (Group 3, *n* = 53). (**B**) **Left:** Event-free survival by mass structure for the whole cohort (*n* = 128) patients enrolled in the study. **Right:** EFS by mass structure for the cohort of patients with MIBG-positive masses (Group 1 *n* = 42). (**C**) **Left:** Resection-free survival for the whole cohort (*n* = 128) patients enrolled in the study. **Right:** RFS for three cohorts of patients based on MIBG procedure: positive (Group 1, *n*= 42), negative (Group 2, *n* = 33) and patients with MIBG not done (Group 3, *n* = 53). (**D**) **Left:** Resection-free survival by mass structure for the whole cohort (*n* = 128) patients enrolled in the study. **Right:** RFS by mass structure for the cohort of patients with MIBG-positive masses (Group 1, *n* = 42).

OS for all children was 100% and EFS at 1 and 3 years was 88% (CI, 82.6 to 93.9) and 87.1% (CI 81.4 to 93.2%), respectively. Group 1 (MIBG+) (N = 42) showed 1- and 3-year EFS rates of 78.5% and 78.5% (CI, 67.0 to 92.0), respectively; Group 2 (MIBG-) (N = 33) showed 97.0% and 97% (CI, 91.3 to 1.0), respectively; and Group 3 (MIBG-ND) (N = 53) showed 90.3% (CI, 82.6 to 98.8), and 88.0% (CI 79.3 to 97.5%), respectively (Figure 2A). There were statistically significant differences at level 0.1 for EFS between Group 1 (MIBG+) and Group 2 (MIBG-) (*p*-value = 0.024, adjusted *p*-value = 0.072).

The predictive impact of mass appearance (cystic, N = 35, 27.3%, mixed N = 46, 35.9% or solid N = 47, 36.7%) on EFS for the whole cohort of sSRM patients was analyzed. EFS at 1 and 3 years was: (a) cystic masses 97.1% (CI: 91.8–100%), and 97.1% (CI: 91.8–100%); (b) mixed masses 93.4% (CI: 86.5–100%), and 90·8% (CI: 82.6–99.9%); and solid masses 76.6% (CI: 65.4–89.7%), and 76.6% (CI: 65.4–89.7%), respectively (Figure 2B). Patients with solid masses showed a lower EFS than patients with cystic masses (*p*-value = 0.013, adjusted *p*-value = 0.039). Analyzing only the MIBG+ group, there was no statistical difference in EFS based on mass structure, possibly due to small sample size. We observed that for this group, there were no events in cystic masses, two in mixed masses and seven in solid masses during the observation period (Figure 2B).

**Figure 3:**
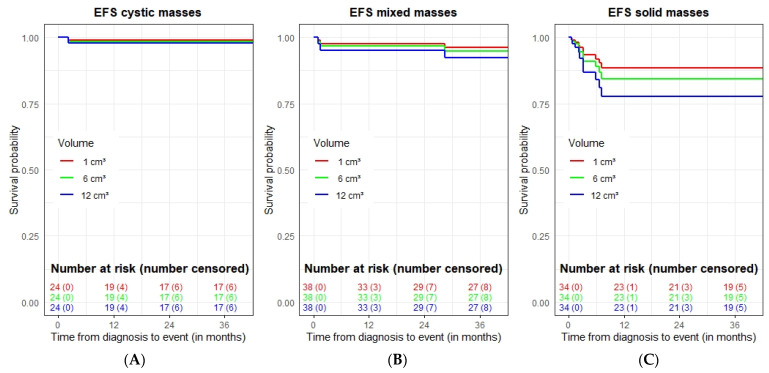
Event-free survival effect of volume and nature of mass for the whole cohort (*n* = 96 available patients with 3D tumor measures from first postnatal US). The predictive evolution of 9 hypothetical patients is shown, combining the three types of mass structure ((**A**) cystic, (**B**) mixed and (**C**) solid) with three volumes: 1 cm^3^, in red; 6 cm^3^, in green, and 12 cm^3^ blue.

We also investigated the combined predictive impact of the volume and structure of the mass on EFS, stratifying the patients according to the structure of the mass and considering the mass volume as a covariate (Figure 3). Three-dimensional (3D) tumor measurements from the first postnatal US (N = 96 with available measurements) were used to construct a COX regression model. The results show that the mass volume is a risk factor for inferior EFS (*p*-value < 0.001) with a greater impact for patients with solid sSRMs. Patients with larger solid masses tend to have an inferior outcome, although statistical significance was not reached.

### 3.2. Surgery

Surgery was performed in 39 patients (30.5%), 18 during the 48-week observation period and 21 afterwards (17 patients aged 12–18 months and 4 patients aged 18–30 months). Thirty-six masses were completely excised, one was incompletely excised and two were only biopsied (Table 4). Neuroblastoma was confirmed in 29 patients (74.4%). Two non-life-threatening surgical complications were observed in two cases: one with acute hemorrhage, and one with an acute renal ischemia with subsequent resolution to normal renal function.

In the 18 patients who had a surgical excision during the observation period, the indications were an increase of >40% in mass size (N = 11), development of distant metastases (N = 5) and size increase of <40% (N = 1) or mass persistence (N = 1). Pathology confirmed neuroblastomas in 16 cases (11 solid, 1 cystic, and 4 mixed masses) and there was a case each of subdiaphragmatic extralobar pulmonary sequestration (solid mass), and a bronchogenic cyst (cystic mass). The median mass diameter prior to surgery was 4.7 cm (range, 1.7–9.0). Twenty-one children were operated on after the 48-week observation period and the diagnoses were: neuroblastoma (N = 13, 5 cystic, 5 solid and, 3 mixed masses), ganglioneuroblastoma intermixed (N = 1, solid mass), ganglioneuroma (N = 1, cystic mass), bronchogenic cyst (N = 2, mixed masses), non-viable tumor lesion (N = 1, solid mass), liver hemangioendothelioma (N = 1, mixed mass), suprarenal gland with fibromyxoid stroma nodular area (N = 1, solid mass), and necrotic tumor (N = 1, cystic mass). The median mass diameter by US prior to surgery was 1.5 cm (range, 0–3.7). For the whole study cohort, the 3-year resection free survival (RFS) rate was 66.1 % (Figure 2C).

The resection rate according to sSRM consistency by US was the following: 20 of 47 (42.5%) solid masses were excised (12 during the observation period and 8 thereafter), 9 of 46 (19.6%) mixed masses were excised (4 during the observation and 5 thereafter), and 9 of 35 (25.7%) cystic masses were excised (2 during observation and 7 thereafter). Analysis of RFS for all patients demonstrated that solid masses were operated on more frequently than cystic masses (*p*-value = 0.028, adjusted *p*-value = 0.084) (Figure 2D). There was no statistical difference in RFS based on mass structure in Group 1 (MIBG+) only patients, possibly due to the smaller sample number.

### 3.3. Spontaneous Evolution of sSRM

**Figure 4:**
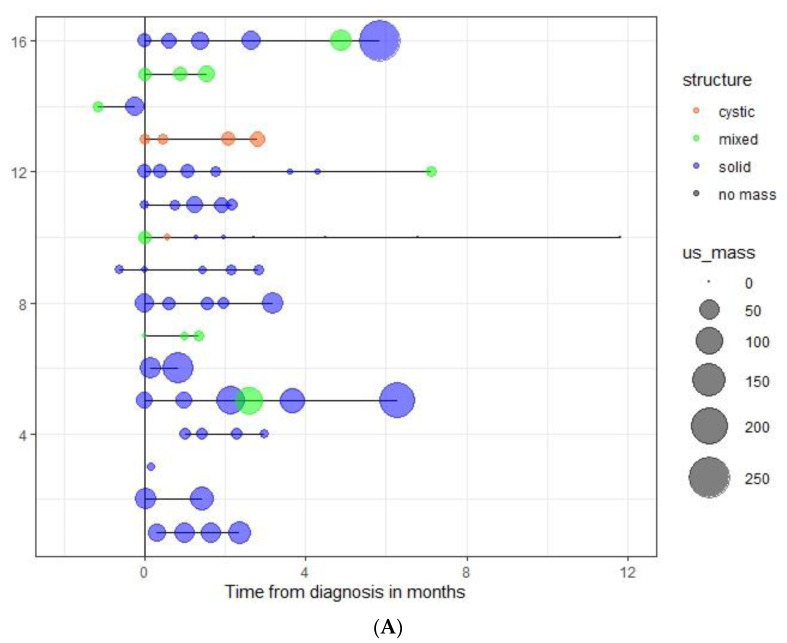

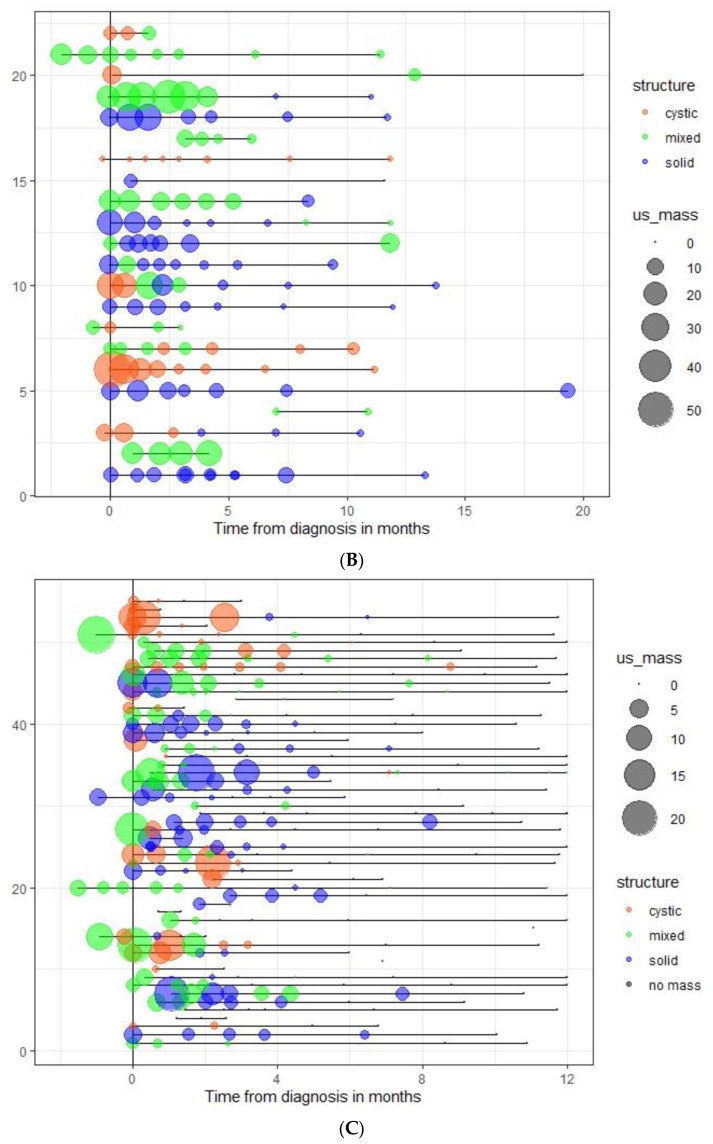
Swimmer/Bubble combined plot graph of patients with a SRM who underwent surgery and had (**A**) or did not have (**B**) an event. (**C**) Patients who achieved complete remission during the 48-week observation period. The plot displays evolution of the mass over time until surgery. Each patient is represented as a single horizontal line. Bubbles depict tumor volume (3D measurements in ml) for each US imaging monitoring point. Type of mass is color coded: orange (cystic), green (mixed) and blue (solid).

(A) For the whole cohort: 51 of 128 (39.8%) masses regressed completely prior to one year from diagnosis, whereas 13 of 128 patients (10.2%) regressed after one year (Figure S2). The regression kinetics analysis was assessed for 64 patients who had a complete follow-up for the 48 weeks observation period. The median patient age at the time of complete regression was 29.8 weeks (range, 3.6–180.8 weeks) and the median time from diagnosis to complete regression was 27.8 weeks.

(B) For the Group 1 MIBG+ patients: 11 of 42 (26.2%) masses regressed completely before one year from diagnosis, compared to 5 (11.9%) afterwards. The regression kinetics analysis was assessed for 11 patients in Group 1 (MIBG+) achieving complete mass regression. The median patient age at the time of mass regression was 48.0 weeks (range, 23.3–180.5 weeks). Median time to complete regression was 45.3 weeks.

(C) Bubble and Swimmer plots. Figure 4 depicts the evolution (size and structure) of the mass at presentation and each serial US evaluation time points for all the operated cases. These visual analytics show that the vast majority of patients who had an event were imaged with a solid mass during the serial monitoring.

## 4. Discussion

Small suprarenal masses have a range of diagnostic possibilities, most commonly being neuroblastomas which at this age group usually have excellent prognosis and often regress spontaneously. Patients with sSRMs can be diagnosed prenatally and their presence can trigger potentially unnecessary surgical resections in otherwise healthy neonates.

The current study confirmed an excellent survival and outcome of these children with initial watchful observation. We showed that solid masses underwent surgery most frequently and were more likely to be neuroblastomas, while cystic and mixed masses were more prone to spontaneous regression, with fewer events and a better outcome. These data are slightly different from the results reported by Nutchern and colleagues who did not find differences in event rates between solid and cystic masses [18]. Since serial US examinations are easy to perform and are now standard practice, we consider this difference in the evolution and outcome between solid and cystic masses to be an important message for pediatricians and obstetricians caring for fetuses and neonates and consulting their caregivers.

Although MIBG has been the gold-standard test for diagnosis and staging of neuroblastoma since 1988, 41% of our population did not have an MIBG performed (local physician-parent shared decision) [22]. We are aware that pediatric oncologists can be worried about radioisotope exposure in very small babies as well as the need for sedation and adequate thyroid blockade. In addition, there are increasing data on the favorable biological behavior and outcome for this patient population which contributes to physicians trying to avoid MIBG scans [2,3,23].

We classified patients according to MIBG uptake to evaluate whether this should be a compulsory examination for sSRMs. In MIBG+ patients, observed events were evolution to L2 or MS neuroblastoma, resulting in EFS of about 80% and confirming previously published data [18]. In MIBG-patients, the only mass with volume increase proved to be a bronchogenic cyst. Patients who did not have an MIBG scan had high 3-year EFS and no deaths; however, they had 6 neuroblastoma related events (evolution to higher neuroblastoma stage, L2, or MS). In the whole cohort (128 patients), five infants progressed to MS neuroblastoma; of note, only one had MIBG imaging performed according to protocol and with positive uptake. We recommend that, except for cases with significant mass regression or disappearance before 3 months of age, MIBG must be considered and undertaken, as it is an essential examination for the definitive diagnosis of sSRMs.

Urine catecholamines were not found to be reliable for neuroblastoma diagnosis. MYCN amplification detection in plasma could be useful, to select out high-risk cases but we observed very low quantities of circulating DNA in the samples drawn and no MYCN amplification in any infant sample. These results may be explained by the small size of tumors and they are in line with previous report showing that the quantity of circulating DNA increases with disease stage [24]. Only one case, with resection at 48 weeks due to mass persistence, proved to be MYCN amplified neuroblastoma and of note the plasma evaluated at diagnosis did not confirm MYCN amplification. This can be interpreted either as failure in methodology and detection limits as observed previously, or it can be related to clonal evolution within the tumor over time [21].

In summary, until more sophisticated biomarkers are developed, we still have to rely on MIBG whole body scintigraphy as a specific and sensitive test for the non-surgical diagnosis of suprarenal neuroblastoma.

Surgery was performed in almost one-third of the patients, mostly due to persistence of the mass or increase in mass volume. Our study clearly shows that surgery can be avoided in two thirds of cases and safely postponed in the other third, avoiding surgery in a neonate and allowing for better surgical conditions and possibly for minimally invasive surgery. The absence of acute irreversible complications from surgery was notable, with no long-term adverse events documented.

Our study was not designed to investigate the psychological impact of attentive monitoring on parents and carers, but it should be considered. They had to attend for frequent visits, in addition to facing ongoing concerns about possibility of their child’s mass (of unknown pathology) increasing in size when surgery was not electively performed at initial detection of the mass. We do not know how many parents might have refused enrolling in this initial observational study if they had been aware of the effects of continued observation (and the associated worries) and they might have elected for immediate surgical intervention to likely definitively solve the problem. However, since the publication by the Food and Drug Administration of an alert on the risks of general anesthesia and sedation drugs on the developing brain of children under three years of age, it is likely that the idea of avoiding surgery or deferring it, may be well accepted by the parents [25].

Patient follow-up was quite complete during the planned 48-week observation period (6% lost to follow-up) but rather incomplete after that, with 20% of cases reported as lost to follow-up at 3 years, due to patients being discharged to local centers to facilitate hospital visits being closer to home.

The present study describes the natural history of sSRMs based on serial imaging. Our analysis highlighted that the majority of masses regressed within the first 6–7 months from diagnosis and some regressed very early. In fact, while 128 masses were detected by postnatal US (median 8.5 days of life), only 81 were demonstrated with the first MRI imaging (median: 36 days of life). The final pathological diagnosis of these 47 cases is not known, but no events occurred. The median time needed for complete tumor regression in 11 MIBG+ children was almost 1 year. These observations should be taken into account when designing new studies and consideration should be given to delaying the MIBG scanning to after 6 months of age or performing it only for solid masses.

## 5. Conclusions

In conclusion, we confirmed that close observation of sSRM with serial radiological monitoring is safe with no negative impact on a very favorable outcome. Surgery can be avoided or delayed in most infants with sSRMs. Nevertheless, solid (rather than cystic) sSRMs should be followed, including with an early MRI and MIBG scan, and the mass resected before the end of the first year if regression is not confirmed. We suggest that in cases of cystic and mixed sSRMs, when serial US monitoring shows regression of the mass and in the absence of signs of progression, MIBG scanning could be postponed or even avoided and surgery may not be needed.

## Figures and Tables

**Table 1:** Observation: Procedures.

Follow-Up (Weeks)	0	3	6	9	12	18	30	48
US	+	+	+	+	+	+	+	+
Catecholamines	+	+	+	+	+	+	+	+
MRI	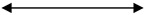					+		+
MIBG	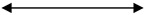							

Week 12: If regression has been observed, US and catecholamines can be performed at weeks 18, 30 and 48. If regression has not been observed, US and catecholamines can be performed monthly until week 48.

**Table 2:** Patient Characteristics.

	Number of Patients	% of Total
**Total**	128	
Males	78	60.9
Females	50	39.1
**Median age at diagnosis (days)**	3	
Range	0–87	
**Time of detection**		
Prenatal	54	42.2
Postnatal	74	57.8
**Location of mass by MRI (N = 82)**		
Suprarenal	75	91.5
Abdominal non-suprarenal	2	2.4
Regressed *	5	6.1
**Structure of mass by MRI (N = 77)**		
Cystic	22	28.6
Solid	28	36.4
Mixed	27	35
**Structure of mass by US (N = 128)**		
Cystic	35	27.3
Solid	47	36.7
Mixed	46	35.9
**Vascularization of mass by US (N = 128)**		
Present	35	37.3
Absent	55	43
Not evaluable	36	28.1
Not evaluated	2	1.6

* Completed Regression by time of MRI.

**Table 3:** Characteristics of patients with event.

Patient Nº	Sex	Time to Event	Age at Diagnosis	Type of Event	Time to Surgery	Surgery	Final Diagnosis	MIGB at Diagnosis	MYCN *	Mass Structure at Diagnosis
A	F	22	0	MS	28	Complete excision	NB	ND	ND	Solid
B	F	26	2	Size increase	40	Complete excision	NB	Positive	Negative	Solid
C	F	30	1	Size increase	62	Complete excision	NB	Positive	ND	Mixed
D	M	43	18	Size increase	50	Complete excision	NB	Positive	Negative	Mixed
E	F	47	0	Size increase	51	Complete excision	NB	ND	ND	Solid
F	M	47	39	MS	60	Biopsy only > 50% residual tumor	NB	ND	ND	Mixed
G	F	64	0	Size increase	205	Complete excision	Bronchogenic cyst	Negative	Negative	Cystic
H	M	66	3	MS	72	Complete excision	NB	ND	ND	Solid
I	F	72	72	Size increase	83	Complete excision	NB	Positive	Negative	Solid
J	F	97	44	Size increase	131	Complete excision	NB	Positive	Negative	Solid
K	F	98	79	Size increase	98	Excision with minimal residual tumor (<5% or <5 mL)	NB	Positive	Negative	Solid
L	F	109	0	MS	140	Complete excision	NB	Positive	ND	Solid
M	F	178	30	MS	179	Biopsy only > 50% residual tumor	NB	ND	ND	Solid
N	M	205	29	Size increase	205	Complete excision	NB	Positive	Negative	Solid
O	M	217	36	Size increase	302	Complete excision	NB	Positive	Negative	Solid
P	F	865	60	Size increase	882	Complete excision	NB	ND	Negative	Mixed

F: female; M: male; Size increase indicates an increase in tumor volume 40%; ND indicates not done; * MYCN amplification status analyzed in plasma at diagnosis, MYCN: cell free DNA analysis for MYCN amplification in plasma. All times are in days.

**Table 4:** Summary of patients who underwent resection. In grey, cases who had an event (MS or increased ≥ 40% in tumor volume). In white, cases who had a persisting mass at the end of the 48 weeks observation period and were referred to surgery. Urine catecholamines (VMA/HVA/DOPA) and LDH are shown in red in values above the 2× the upper normal value (>2 u.n.v.) and in green if normal (≤2 u.n.v). CR; complete resection; ND: not done; GNB; ganglioneuroblastoma; GN: ganglioneuroma: NB; neuroblastoma.

Patient Nº	Months between Diagnosis and Surgery	MIGB at Diagnosis (Y (+, −, ND)	Urine Catecholamines and LDH at Diagnosis	Structure of the Mass at Diagnosis	Surgery Outcome	Final Diagnosis	Indication for Surgery
1	0.9	ND	ND	Solid	CR	NB	MS
2	1.3	(+)	**LDH/VMA/HVA**	Solid	CR	NB	size increased
3	1.7	ND	LDH/HVA/**VMA**	Solid	CR	NB	size increased
4	1.7	(+)	**LDH**/VMA/HVA/DOPA/COR	Mixed	CR	NB	size increased
5	2.0	ND	LDH/VMA/HVA	Mixed	Biopsy *	NB	MS
6	2.1	(+)	LDH/VMA/HVA	Mixed	CR	NB	size increased
7	2.4	ND	LDH/**VMA**	Solid	CR	NB	MS
8	2.8	(+)	LDH/HVA/**VMA**	Solid	CR	NB	size increased
9	3.3	(+)	LDH/VMA/HVA	Solid	Excision with residue **	NB	size increased
10	4.4	(+)	VMA/HVA	Solid	CR	NB	size increased
11	4.7	(+)	VMA/HVA/DOPA	Solid	CR	NB	MS
12	4.7	(+)	LDH/VMA/HVA/DOPA	Cystic	CR	NB	size increased <40%
13	6.0	ND	**LDH**/VMA/HVA	Solid	Biopsy *	NB	MS
14	6.8	(−)	LDH/VMA/HVA/DOPA/COR	Cystic	CR	bronchogenis cyst	size increased
15	6.8	(+)	LDH/HVA/**VMA**	Solid	CR	NB	size increased
16	10.0	(+)	LDH/VMA/HVA	Solid	CR	Subdiaphragmatic extralobar pulmonary sequestration	persisting mass
17	10.1	(+)	LDH/VMA/HVA/DOPA/COR	Solid	CR	NB	size increased
18	11.0	ND	LDH/VMA/HVA	Mixed	CR	NB	persisting mass
19	12.3	(+)	VMA/HVA	Cystic	CR	NB	persisting mass
20	12.7	(−)	LDH/DOPA	Cystic	CR	NB	persisting mass
21	12.9	(+)	LDH	Solid	CR	suprarenal gland with nodular area composed of fibromyxoid stroma	persisting mass
22	13.2	ND	LDH/VMA/HVA	Solid	CR	NB	persisting mass
23	13.2	ND	LDH/VMA	Mixed	CR	Hemangioendothelioma of the liver	persisting mass
24	13.7	(+)	VMA/HVA/DOPA	Mixed	CR	NB	persisting mass
25	14.3	(−)	ND	Cystic	CR	NB	persisting mass
26	14.5	(−)	LDH/VMA/HVA/COR	Cystic	CR	NB	persisting mass
27	14.7	(−)	LDH	Mixed	CR	bronchogenis cyst	persisting mass
28	14.8	ND	VMA/HVA	Solid	CR	NB	persisting mass
29	14.8	(+)	LDH/VMA/DOPA/**HVA**	Solid	CR	NB	persisting mass
30	15.2	(−)	LDH/VMA/HVA/DOPA	Cystic	CR	GN	persisting mass
31	15.3	ND	LDH/VMA/HVA/DOPA/COR	Mixed	CR	bronchogenis cyst	persisting mass
32	15.5	(+)	LDH/VMA	Mixed	CR	NB	persisting mass
33	16.0	(+)	LDH/VMA/HVA/**DOPA**	Cystic	CR	Totally necrotic tumor.No malignancy	persisting mass
34	16.5	(+)	DOPA	Solid	CR	cicatricial lesion without viable tumor (>99.9% necrosis)	persisting mass
35	16.8	(−)	LDH/VMA/HVA/DOPA	Solid	CR	GNB intermixed	persisting mass
36	19.3	ND	VMA/HVA	Cystic	CR	NB	persisting mass
37	24.0	(+)	LDH/VMA/HVA	Solid	CR	NB	persisting mass
38	24.9	(−)	VMA	Solid	CR	NB	persisting mass
39	29.4	ND	LDH/DOPA/COR	Mixed	CR	NB	size increased

* Biopsy only (>50% residual tumor). ** Excision with minimal residual tumor (<5% or <5 mL residual tumor).

## Data Availability

The data are not publicly available due to ethical reasons.

## Supplementary Materials

The following supporting information can be downloaded at: https://www.mdpi.com/article/10.3390/cancers14164007/s1, Figure S1: Concordance between US pre and postnatal; Figure S2: Kinetics modeling of mass regression for: (A) and (B) the whole cohort (*n* = 128) (C) and (D) patients with MIBG-positive masses (*n* = 42). Table S1: List of participating sites and investigators.
