# Supplementary material for: Suprarenal Masses in Very Young Infants: Is It Safe to Watch and Wait? Report of a SIOPEN Observational Study Results
Source: Cancers (Basel). 2022 Aug 19;14(16):4007. doi: 10.3390/cancers14164007 (PMC9406882; doi:10.3390/cancers14164007)
Supplement: Supplementary file 1 [file cancers-14-04007-s001.zip › cancers-1846310-supplementary.pdf]

Table S1: List of participating sites and investigators

SIOPEN participating centres	Principal Investigator	Accrual
Ospedale Bambino Gesu	Aurora Castellano	16
CHU de Grenoble	Dominique Plantaz	7
Clinica di Oncoematologia Pediatrica Padova	Elisabetta Viscardi	7
Istituto Giannina Gaslini	Massimo Conte	7
Azienda Osped. Univ. Consorziale Policlinico Bari	Francesco De Leonardis	5
Centre Oscar Lambret de Lille	Anne-Sophie Defachelles-Thomassin	5
Hopitaux de Marseille La Timone	Carole Coze	5
U.O. di Ematologia Clinica ASL di Pescara	Silvia Pascale	5
Centre Hospitalier Angers	Isabelle Pellier	4
Hôpital D'Enfants de Toulouse	Herve Rubie	4
Istituto Nazionale Tumori di Milano	Marta Podda	4
Ospedale G. Salesi	Paola Coccia	4
CHU Rouen	Jean-Pierre Vannier	3
Hospital Santa Creu i Sant Pau	Montserrat Torrent	3
Hospital Universitario i Politecnic La Fe	Adela Cañete	3
Institut Gustave Roussy	Dominique Valteau-Couannet	3
Hospital de Cruces	Ricardo López-Almaraz	3
Ospedale dei bambini, Palermo	Paolo D'Angelo	3
Santobono-Pausilipon	Serena Ruotolo	3
CHR de Nantes	Estelle Thebaud	2
CHR Hôpital Sud de rennes	Sophie Taque	2
Gemelli Hospital of Rome	Stefano Mastrangelo	2
Hôpital de L'Archet Nice	Marilyne Poiree	2
Hopital Americain de Reims	Martine Munzer	2
Hospital Central de Asturias	Jose Antonio Villegas	2
Hospital Clinico Universitario Valencia	Francisco Mares	2
Hospital Materno Infantil Vall d'Hebron	Soledad Gallego	2
Hospital Virgen del Rocio	Catalina Márquez	2
Institut Curie	Gudrun Schleiermacher	2
Ospedali Riuniti di Bergamo	Massimo Provenzi	2
Policlinico Universitario Catania	Andrea Di Cataldo	2
Schneider Children's Medical Center of Israel	Shifra Ash and Sivan Achituv	2
Casa Sollievo della Sofferenza	Lucia Miglionico	1
CHR de Besancon	Véronique Laithier	1
CHU Montpellier Hôpital Arnaud Villeneuve	Nicolas Sirvent	1
CHU-Saint Etienne	Jean-Louis Stephan	1
Dana Children's Hosp., Suraski Tel-Aviv Med. Cent.	Sivan Achituv	1
Hopital Jean Bernard La Miletrie Poitiers	Frédéric Millot	1
Hospital General Universitario de Alicante	Carlos Esquembre	1
Landes-Kinderklinik Linz	Georg Ebetsberger-Dachs	1
O.I.R.M. - S. Anna	Maurizio Bianchi	1
University Hospital Gent	Geneviève Laureys	1

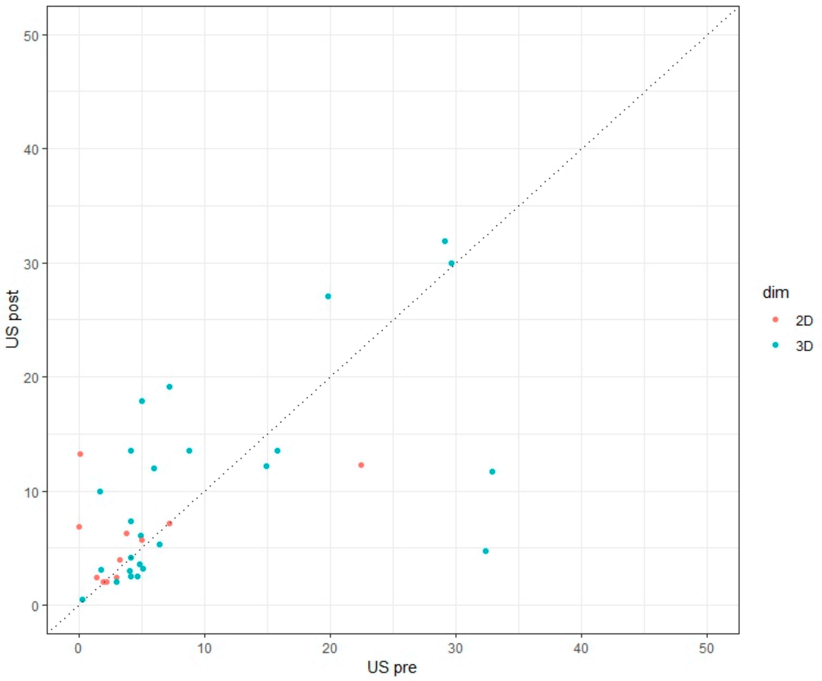

Figure S1. Concordance between US pre and postnatal.

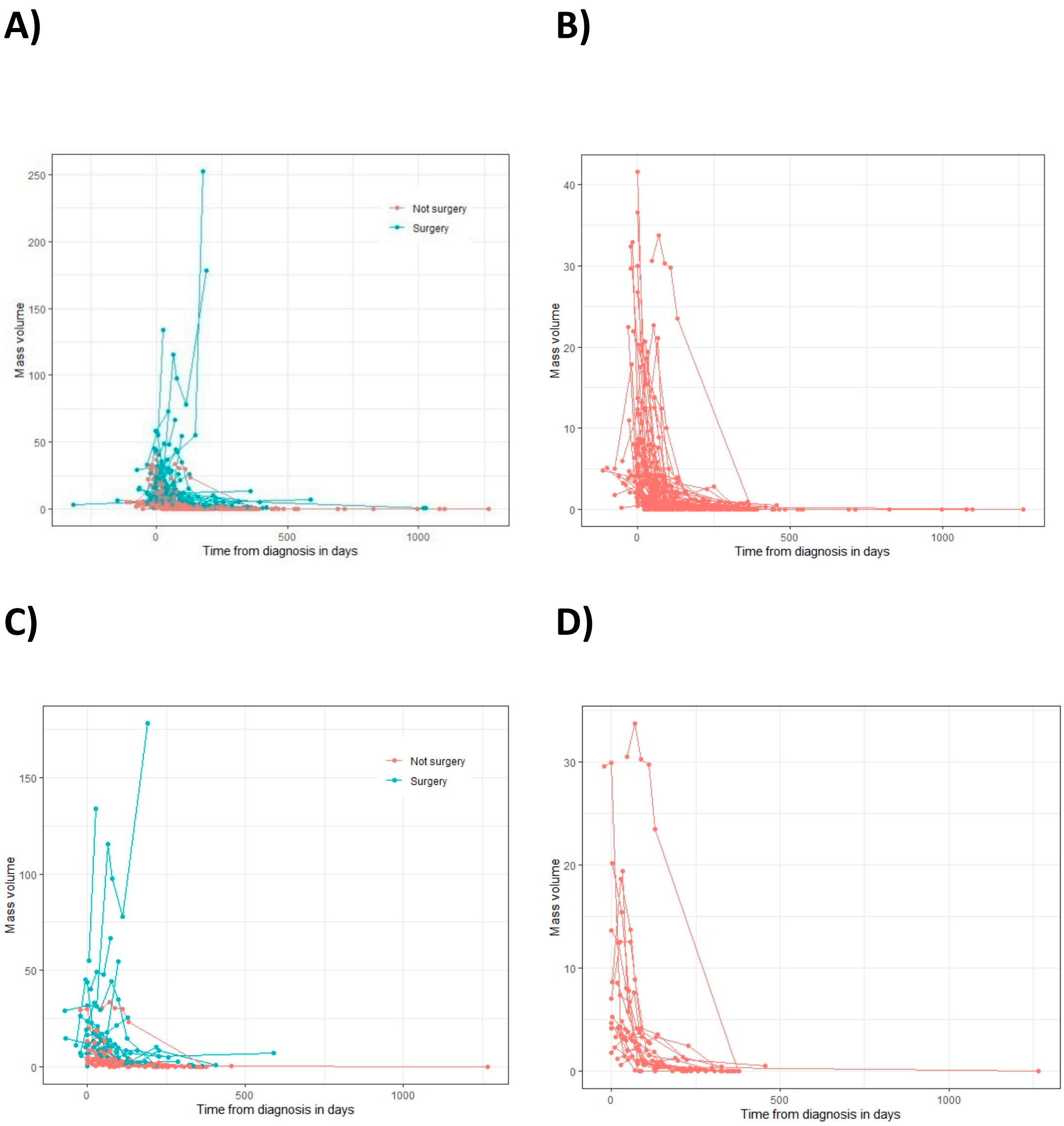

Figure S2. Kinetics modeling of mass regression for: A and B) the whole cohort (n=128) C) and D) patients with MIBG positive (n=42). Blue dots indicate data from patients who underwent surgery. Red dots indicate data from patients who did not undergo surgery. Note that the x-axis represents time from diagnosis, and the y-axis the tumor volume in ml.
